# Cellular density‐dependent increases in HIF‐1α compete with c‐Myc to down‐regulate human EP4 receptor promoter activity through Sp‐1‐binding region

**DOI:** 10.1002/prp2.441

**Published:** 2018-11-11

**Authors:** Naofumi Seira, Kazuyuki Yamagata, Keijo Fukushima, Yumi Araki, Naoki Kurata, Naoki Yanagisawa, Masato Mashimo, Hiroyuki Nakamura, John W. Regan, Toshihiko Murayama, Hiromichi Fujino

**Affiliations:** ^1^ Laboratory of Chemical Pharmacology Graduate School of Pharmaceutical Sciences Chiba University Chuo‐ku Chiba Japan; ^2^ Department of Pharmacology for Life Sciences Graduate School of Pharmaceutical Sciences & Graduate School of Biomedical Sciences Tokushima University Tokushima Japan; ^3^ Laboratory of Pharmacology Faculty of Pharmaceutical Sciences Doshisha Women's College of Liberal Arts Kyotanabe, Kyoto Japan; ^4^ Department of Pharmacology & Toxicology College of Pharmacy The University of Arizona Tucson Arizona

**Keywords:** c‐Myc, colorectal cancer, EP4 receptors, HIF‐1α, homeostasis, Sp‐1

## Abstract

The up‐regulated expression of E‐type prostanoid (EP) 4 receptors has been implicated in carcinogenesis; however, the expression of EP4 receptors has also been reported to be weaker in tumor tissues than in normal tissues. Indeed, EP4 receptors have been suggested to play a role in the maintenance of colorectal homeostasis. This study aimed to examine the underlying mechanisms/reasons for why inconsistent findings have been reported regarding EP4 receptor expression levels in homeostasis and carcinogenesis by focusing on cellular densities. Thus, the human colon cancer HCA‐7 cells, which retain some functional features of normal epithelia, and luciferase reporter genes containing wild‐type or mutated EP4 receptor promoters were used for elucidating the cellular density‐dependent mechanisms about the regulation of EP4 receptor expression. In silico analysis was also utilized for confirming the relevance of the findings with respect to colon cancer development. We here demonstrated that the expression of EP4 receptors was up‐regulated by c‐Myc by binding to Sp‐1 under low cellular density conditions, but was down‐regulated under high cellular density conditions via the increase in the expression levels of HIF‐1α protein, which may pull out c‐Myc and Sp‐1 from DNA‐binding. The tightly regulated EP4 receptor expression mechanism may be a critical system for maintaining homeostasis in normal colorectal epithelial cells. Therefore, once the system is altered, possibly due to the transient overexpression of EP4 receptors, it may result in aberrant cellular proliferation and transformation to cancerous phenotypes. However, at the point, EP4 receptors themselves and their mediated homeostasis would be no longer required.

**Abbreviations**
EP: E‐type prostanoid
COX‐2: cyclooxygenase‐2
PGE_2_: prostaglandin E_2_
HIF: hypoxia‐inducible factor
DMEM: Dulbecco's modified Eagle's medium
FBS: fetal bovine serum
HRE: hypoxia response element
WT: wild‐type
PCR: polymerase chain reaction
ChIP: chromatin immunoprecipitation
BSA: bovine serum albumin
ANOVA: analysis of variance
UCSC: The University of California Santa Cruz
VEGF: vascular endothelial growth factor
VEGFR: VEGF receptor
GLUT: glucose transporter
VHL: von Hippel‐Lindau
PHD: prolyl hydroxylase domain‐containing enzyme
EGLN: Egl‐9 family hypoxia‐inducible factor
FIH1: factor‐inhibiting HIF‐1α
HIF1AN: HIF‐1α inhibitor
COMMD: copper metabolism domain containing
HSP: heat shock protein
CBP: cAMP response element‐binding protein‐binding protein
CITED: CBP/p300‐interacting transactivator.

## 1 INTRODUCTION

### 1.1 E‐type prostanoid 4 receptors; their involvement in colorectal cancer and homeostasis

Increases in the levels of cyclooxygenase‐2 (COX‐2) and prostaglandin E_2_ (PGE_2_) are well‐known biomarkers for the early stage of colorectal cancer.^1^, ^2^ The up‐regulation of COX‐2 expression is associated with the activation of E‐type prostanoid (EP4) receptors,^3^ and the EP4 receptor‐mediated signaling pathway is also associated with increases in cell motility and proliferation^4^ in human colon cancer cells. Moreover, the up‐regulated expressions of EP4 receptors,^5^, ^6^ and PGE_2_ synthase^7^ have been reported during the progression of colorectal cancer, and increased expression levels of PGE_2_ synthase were shown to be mediated by the activation of EP4 receptors.^8^ Thus, the activation of EP4 receptors establishes a positive feedback loop that may drive the expression of COX‐2 and PGE_2_ synthase, followed by the synthesis of PGE_2_; that is, EP4 receptors are considered to play functional roles in the malignancy of colorectal cancer.^9^

While EP4 receptors are generally implicated in colorectal carcinogenesis, they may also be involved in maintaining gastrointestinal homeostasis.^10^ Normal colorectal epithelial cells have been shown to express EP4 receptors,^10^ with strong expression in lateral crypt epithelia,^11^ and have a turnover cycle of 3‐5 days.^12^ Thus, stem cells at the crypt bottom generate epithelial progenitor cells, these cells proliferate, migrate, and differentiate to the intestinal lumen, and then undergo apoptosis and/or extrusion into the lumen over 3‐5 days.^13^ New epithelial progenitor cells have been reported to accumulate *β*‐catenin and stimulate *β*‐catenin‐mediated transcriptional activity, which induces cells to proliferate and migrate until they reach the midcrypt region. At the midcrypt region, cells inhibit *β*‐catenin‐mediated activity, which leads to cell cycle arrest and differentiation, followed by apoptosis and/or extrusion when cells reach the lumen surface.^13^ Thus, *β*‐catenin‐mediated signaling may function as the dominant switch between the proliferation and differentiation of colorectal epithelial cells.

Although the up‐regulated expression of EP4 receptors has been demonstrated during colorectal development, another study showed that the mRNA expression levels of EP4 receptors were higher in normal colon tissues than in cancer tissues.^14^ The expression levels of EP4 receptors were previously reported to be regulated by Sp‐1, a zinc finger transcription factor, binding to two Sp‐1‐binding sites in the human EP4 receptor promoter region from −197 to −160 bp.^15^ However, another transcription factor, which is induced under hypoxic conditions, hypoxia‐inducible factor (HIF)‐1α, was shown to down‐regulate the expression of EP4 receptors when human colon cancer HCA‐7 cells were cultured under high cellular density conditions, the environment of the cells that is equivalent to over proliferated conditions.^16^ Thus, the cellular density‐dependent induction of HIF‐1α protein expression down‐regulates the expression of EP4 receptors in HCA‐7 cells.^16^ Since the expression of HIF‐1α is known to be up‐regulated and correlates with cancer malignancy,^17^, ^18^, ^19^ massively proliferating cancer cells may exhibit the decrease in the expression of EP4 receptors.

### 1.2 Translational regulation of the promoter by HIF‐1α, c‐Myc, and Sp‐1

The activation of *β*‐catenin‐mediated signaling was previously reported following the stimulation of EP4 receptors with PGE_2_.^20^ The proliferation of colorectal cancer epithelial cells has also been suggested to be mediated by the effects of c‐Myc induced by *β*‐catenin‐mediated signaling.^13^ Therefore, the c‐Myc‐mediated proliferation of cells may be regulated via EP4 receptor activation. HIF‐1α has been reported to bind to Sp‐1 by displacing c‐Myc from Sp‐1‐binding sites to reduce the DNA mismatch repair system in colon cancer cells.^21^, ^22^ Thus, regarding the regulation of EP4 receptor expression, c‐Myc has been implicated in the up‐regulation of receptors, whereas HIF‐1α exerts the opposite effects, with the involvement of Sp‐1.

We herein demonstrate that the expression of EP4 receptors is tightly regulated by c‐Myc and HIF‐1α by binding to Sp‐1 as cellular density‐dependently in HCA‐7 cells. This tight regulation of EP4 receptor expression by c‐Myc and HIF‐1α may be an essential system for maintaining homeostasis in normal colorectal epithelial cells. However, once the system is altered, it may cause aberrant cellular proliferation, the transformation from normal to cancerous phenotypes, which represents the trigger for the early stage of colorectal carcinogenesis.

## 2 MATERIALS AND METHODS

### 2.1 Cell culture and materials

The human colon cancer cell line HCA‐7 was cultured in Dulbecco's modified Eagle's medium (DMEM; Nacalai Tesque, Kyoto, Japan) containing 10% fetal bovine serum (FBS) (Thermo Fisher Scientific, Waltham MA), 100 UI/mL penicillin (Meiji Seika, Tokyo, Japan), and 100 μg/mL streptomycin (Meiji Seika) in 5% CO_2_ at 37°C. All materials were obtained from Wako Pure Chemical (Osaka, Japan) unless otherwise stated.

### 2.2 Construction of deletion and mutated EP4 promoter luciferase reporter plasmids

Deletion mutants, and point mutations introduced into the hypoxia response element (HRE) region, of human EP4 receptor promoter luciferase reporter plasmids were constructed using the human EP4 receptor promoter luciferase reporter plasmid, wild‐type (WT) (−1238/+1),^16^ as a template. In order to construct the deletion mutants of human EP4 receptor promoter luciferase plasmids, primers with the following sequences were used for polymerase chain reaction (PCR) amplification: deletion 1 (−789/+1): 5′‐GGGGCTAGCCCAAGGCTCCACCTCTCTCCAAAGCCGCAA‐3′ (sense), deletion 2 (−473/+1): 5′‐GGGGCTAGCTCGGCCAACCCTAGGTAGAATCCTAAAAC‐3′ (sense), deletion 3 (−197/+1); 5′‐GGGGCTAGCGCCCAGCCCCGCCCCAGCCCAGACACCGCCC‐3′, deletion 4 (−160/+1); 5′‐GGGGCTAGCAGTCTTCCCTGCGGC‐3′ (sense). The sequence of the antisense primer for all deleted plasmids was as follows: 5′‐GGAAGCTTTGGAGCTCGCGTGCTGCGGCCTTTC‐3′. The products of the deleted EP4 receptor promoter by PCR were digested with Nhe I (Takara Bio, Shiga, Japan) and Hind III (Takara Bio) restriction enzymes and then purified and ligated into pGL3‐basic vectors (Promega, Madison, WI). The point mutations introduced in HRE containing the human EP4 receptor promoter luciferase plasmid were amplified by PCR using the primers 5′‐TCCGCACCCCCGAGGGAATGAAAACCACGGGAGCC‐3′ (sense) and 5′‐GGCTCCCGTGGTTTTCATTCCCTCGGGGGTGCGGA‐3′. The deletion 3 (−197/+1) luciferase plasmids, in which point mutations were introduced at each or both Sp‐1‐binding sites, were constructed by PCR using the following primers: mut‐A‐del 3: 5′‐GCCCAGCCCTTCCCCAGCCCA‐3′ (sense) and 5′‐TGGGCTGGGGAAGGGCTGGGC‐3′ (antisense), mut‐B‐del 3: 5′‐GCCCAGACACTTCCCCCCGCCA‐3′ (sense) and 5′‐TGGCGGGGGGAAGTGTCTGGGC‐3′ (antisense) and both primer sets for mut‐A,B‐del 3.^15^ The PCR products of each or both Sp‐1 site point mutations introduced into del 3 plasmids were digested with Dpn I (Takara Bio) and self‐ligated. Each construct was sequenced and verified.

### 2.3 Luciferase assay

Cells were cultured under low (2 × 10^5^ cells/each well) and high (2 × 10^6^ cells/each well) cellular density conditions in 6‐well plates, and culture medium was replaced with Opti‐MEM I (Thermo Fisher Scientific) containing 100 UI/mL penicillin and 100 μg/mL streptomycin. Cells were transiently transfected with 10 μg/each well of firefly WT (−1238/+1), deleted or mutated reporter luciferase plasmids, and with 10 ng/each well of renilla luciferase control plasmids, pRL‐CMV (Promega) using Polyethylenimine MAX (MW 40 000) (Polysciences, Warrington, PA) reagent. After approximately 6 hours, the transfection reagent was removed by a medium change using DMEM containing 10% FBS, and cells were incubated for a further 16 hours. To measure the effects of HIF‐1α and c‐Myc on EP4 receptor promoter activity, cells were cultured under low‐density conditions, and culture medium was replaced with Opti‐MEM I containing 100 UI/mL penicillin and 100 μg/mL streptomycin. In HIF‐1α and c‐Myc overexpression experiments, cells were cultured under low cellular density conditions in 12‐well plates, and were transiently transfected with 2 μg/each well of firefly WT (−1238/+1) EP4 receptor promoter luciferase plasmids, 3 ng/each well of pRL‐CMV renilla luciferase control plasmids, and either the pHA‐N1 vector, which was created by replacing EGFP with HA in the pEGFP‐N1 vector (Clontech Laboratories, Mountain View, CA), HA‐HIF1alpha‐pcDNA3 was a gift from William Kaelin (Addgene plasmid #18949) 23 or the pCMFlag_hsc‐Myc (RDB06671, RIKEN BRC, Saitama, Japan) expression vector, using the same reagents described above, and cells were then incubated for a further 42 hours. In competition assays, HA‐tagged HIF‐1α expression plasmids alone (0.5 μg/each well), or HA‐tagged HIF‐1α expression plasmids (0.5 μg/each well) plus various amounts of FLAG‐tagged c‐Myc expression plasmids (0.05, 0.15, and 0.5 μg/each well, respectively); or FLAG‐tagged c‐Myc expression plasmids alone (0.5 μg/each well), or FLAG‐tagged c‐Myc expression plasmids plus various amounts of HA‐tagged HIF‐1α expression plasmids (0.05, 0.15, and 0.5 μg/each well, respectively), and the total amounts of transfected plasmids were adjusted by adding pHA‐N1 control plasmids to 1 μg/each well. Reporter plasmid‐transfected cells were then lysed and assayed using the Dual luciferase reporter assay system (Promega) according to the manufacturer's instructions with the GL‐200 luminometer (Microtech Nichon, Chiba, Japan). Data were normalized by calculating the ratios of firefly luciferase scores to the corresponding renilla luciferase values.

### 2.4 Chromatin immunoprecipitation (ChIP) assay

HCA‐7 cells were cultured under low‐density (5.5×10^5^ cells/dish) and/or high‐density (5.5 × 10^6^ cells/dish) conditions in a 10‐cm dish, and culture medium was replaced with Opti‐MEM I containing 100 UI/mL penicillin and 100 μg/mL streptomycin. Cells were transiently transfected with 25 μg/dish of WT (−1238/+1) or point mutations‐introduced mut‐HRE (−1238/+1) of EP4 receptor promoter reporter luciferase plasmids using Polyethylenimine MAX (MW 40 000) reagent. After approximately 6 hours, transfection reagents were removed by medium changes and cells were incubated for a further 18 hours. A ChIP analysis was performed using a ChIP assay kit according to the manufacturer's instructions (Merck, Kenilworth, NJ) or Dynabeads Protein G (1004D: Invitrogen, CA). Briefly, cells were fixed with 1% formaldehyde at room temperature for 10 minutes. Fixed cells were scraped into microcentrifuge tubes and lysed in SDS lysis buffer. DNA was sheared to fragments by sonication. Sonicated lysates were immobilized with anti‐Sp‐1 antibody (GTX110593: GeneTex, CA), anti‐c‐Myc antibody (GTX103436: GeneTex), and/or an anti‐HIF‐1α antibody (H1alpha67) (NB100‐105; Novus Biologicals, Littleton, CO). Crosslinked DNA by formaldehyde was released from the antibody‐captured protein‐DNA complex and purified. Captured DNA was then eluted and detected by PCR amplification (C1000 Thermal Cycler, Bio‐Rad) or real‐time PCR amplification (Eco Real‐Time PCR systems, Illumina, San Diego, CA). The region between −1238 and +1 of the human EP4 receptor promoter was amplified using the following primers: 5′‐GGGCTAGCCTGCAGATGGGAAGAGGTTTTTCCAGGAATTTAAA‐3′ (sense), 5′‐GGAAGCTTTGGAGCTCGCGTGCTGCGGCCTTTC‐3′ (antisense) by PCR. The relative recruitment of each Sp‐1, c‐Myc, and HIF‐1α to Sp‐1‐binding consensus motifs was detected by using PowerUp SYBR Green Master Mix (Thermo Fisher Scientific) and the following primers: 5′‐GGGGCTAGCGCCCAGCCCCGCCCCAGCCCAGACACCGCCC‐3′ (sense) 5′‐ GTCCACCTCGATATGTGCATC‐3′ (antisense) from ‐197 of the human EP4 receptor promoter to +104 of the firefly gene body by real‐time PCR.

### 2.5 Western blotting

Regarding the detection of Sp‐1, HIF‐1α, and c‐Myc, HCA‐7 cells were cultured under low (2 × 10^5^ cells/each well), middle (6 × 10^5^ cells/each well), and high (2 × 10^6^ cells/each well) density conditions in 6‐well plates, and culture medium was replaced with Opti‐MEM I containing 100 UI/mL penicillin and 100 μg/mL streptomycin for 16 hours. In the detection of EP4 receptor expression, low or high cellular density‐cultured HCA‐7 cells were treated with 100 nmol/L mithramycin A (Wako, Osaka, Japan) for 16 hours. Cells were then scraped with lysis buffer consisting of 150 μmol/L NaCl, 50 mmol/L Tris‐HCl (pH 8.0), 5 nmol/L EDTA (pH 8.0), 1% Igepal CA‐630 (MP Biomedicals, Aurora, OH), 0.5% sodium deoxycholate, 10 mmol/L sodium fluoride, 10 mmol/L disodium pyrophosphate, 0.1% SDS, 0.1 mmol/L phenylmethylsulfonyl fluoride, 1 mmol/L sodium orthovanadate, 10 μg/mL leupeptin (Sigma, St Louis, MO), and 10 μg/mL aprotinin, and then transferred to microcentrifuge tubes. Samples were rotated at 4°C for 30 min and centrifuged at 16 000*g* for 15 minutes. Aliquots of samples containing 20‐40 μg of protein were electrophoresed on 10% SDS‐polyacrylamide gels and transferred to nitrocellulose membranes as described previously.^16^ Membranes were incubated at room temperature for 1 hour in 5% nonfat milk. Incubations were conducted at 4°C for 16 hours in 5% bovine serum albumin (BSA) (Sigma) containing a 1:1000 dilution of an anti‐human EP4 receptor antibody (101775; Cayman Chemical, Ann Arbor, MI); a 1:1000 dilution of an anti‐Sp1 antibody (sc‐420; Santa Cruz Biotechnology, Santa Cruz, CA); a 1:1000 dilution of an anti‐c‐Myc antibody (sc‐40; Santa Cruz Biotechnology); a 1:1000 dilution of an anti‐HIF‐1α antibody (H1alpha67); or a 1:5000 dilution of an anti‐*β*‐tubulin‐antibody (014‐25041; Wako). After being incubated with primary antibodies, membranes were washed three times and then incubated at room temperature for 2 hours with a 1:10 000 dilution of appropriate secondary antibodies conjugated with horseradish peroxidase under the same blocking conditions as those for the primary antibodies.^16^ After washing three times, immunoreactivity was detected and visualized with ChemiDoc XRS Plus Image Lab (Bio‐Rad Laboratories, Hercules, CA). In order to ensure the equal loading of proteins, membranes were stripped and reprobed with the anti‐*β*‐tubulin antibody under the conditions described above. The intensity of chemiluminescence was measured with ImageJ software (National Institutes of Health, Bethesda, MD).

### 2.6 Cell imaging of a hypoxia probe

Cells were cultured under low and high cellular density conditions in 6‐well plates, and culture medium was replaced with Opti‐MEM I containing 100 UI/mL penicillin and 100 μg/mL streptomycin. The hypoxia probe solution LOX‐1 (Medical & Biological Laboratories, Aichi, Japan) was then added to the medium at a final concentration of 2 μmol/L for 24 hours for visualization with the all‐in‐one fluorescence microscope BZ‐X710 (Keyence, Osaka, Japan). The intensity of fluorescence was measured with ImageJ software.

### 2.7 Statistical analysis

Data are expressed as the mean ± SD, and statistical analyses were performed using Prism 7 for windows or Prism 5 for Mac OS X software (GraphPad Software, La Jolla, CA). The *t* test or multiple comparison tests in the analysis of variance (ANOVA) were used to evaluate three or more independent experiments. Additionally, since the original luciferase counts vary greatly among the experiments because of the intrinsic low transfection efficiency of the HCA‐7 cells, we normalized each control value as 100%. Therefore, the one‐sample *t* test was used to evaluate the experimental means ± SD against the control value (100%). Significance was assumed at *P *< 0.05.

### 2.8 Bioinformatic analysis

The University of California Santa Cruz (UCSC) Xena browser ( http://xena.ucsc.edu) was used to obtain Cancer Genome Atlas Colon and Rectal Cancer data. In order to evaluate the mRNA expression of human EP4 receptors and each HIF‐1α‐related gene in cancer tissues and noncancer tissues, the following paired cancer tissue samples and noncancer tissue samples (32 each) from whole samples based on “patient_id” of clinical data were extracted: 2675, 2682, 2684, 2685, 2686, 3489, 3496, 3511, 3655, 3660, 3662, 3663, 3697, 3712, 3713, 3725, 3731, 3732, 3742, 5654, 5659, 5662, 5665, 5667, 6598, 6599, 6600, 6601, 6603, 6605, 6643, and 6704. Statistical analyses were performed with R (version 3.4.1, http://www.R-project.org). The relationships for the expression of each gene between cancer tissues and noncancer tissues were analyzed using the Mann‐Whitney *U*‐test. Significance was assumed at *P *< 0.05.

## 3 RESULTS

### 3.1 Cellular density‐dependent EP4 receptor promoter activities are mediated by HIF‐1α

**Figure 1:**
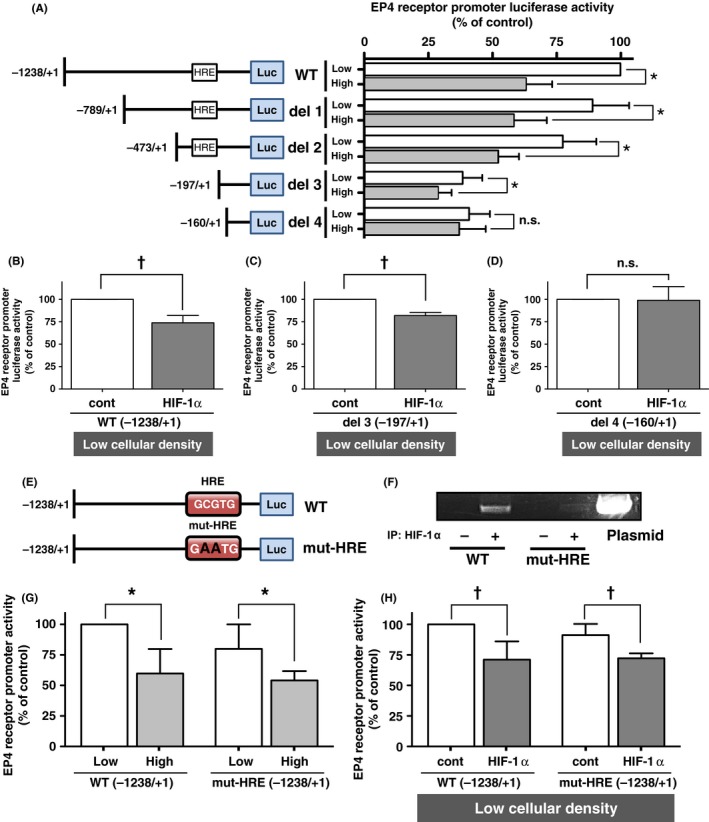
EP4 receptor promoter activities of 5′ deletion mutants and point mutated HRE in HCA‐7 cells. HCA‐7 cells were cultured under low (Low; 2 × 10^5^ cells/each well of 6‐well plate) or high (High; 2 × 10^6^ cells/each well of 6‐well plate) cellular density conditions, and were then transfected with reporter gene plasmids containing WT or each promoter‐mutated plasmid and subjected to the EP4 receptor promoter luciferase assay, as described in the Materials & Methods. (A) The promoter deletion maps and corresponding luciferase activities of each WT (−1238/+1) or the 5′ deletion of EP4 promoter‐containing reporter gene plasmids (del 1 to del 4) obtained from HCA‐7 cells cultured under low or high cellular density conditions. (B, C, D) The luciferase activities of HCA‐7 cells cultured under low cellular density conditions transfected with WT (−1238/+1) (B), del 3 (−197/+1) (C), or del 4 (−160/+1) (D) of each human EP4 receptor promoter plasmid concomitantly with either HA‐control vector plasmids or HA‐tagged HIF‐1α expression plasmids. (E) Schematic maps of WT (−1238/+1) or HRE point mutated WT (mut‐HRE (−1238/+1)) of the EP4 promoter. (F) ChIP assay with anti‐HIF‐1α antibodies performed to verify the binding of HIF‐1α to WT (−1238/+1) or HRE point mutated WT (mut‐HRE (−1238/+1)) of the EP4 promoter in HCA‐7 cells cultured under high cellular density conditions. (G) The luciferase activities of each WT (−1238/+1) or HRE point mutated WT (mut‐HRE (−1238/+1)) of EP4 promoter‐containing reporter gene plasmids obtained from HCA‐7 cells cultured under low or high cellular density conditions. (H) The luciferase activities of HCA‐7 cells cultured under low cellular density conditions transfected either with WT (−1238/+1) or HRE point mutated WT (mut‐HRE (−1238/+1)) of EP4 promoter‐containing reporter gene plasmids concomitantly with either HA‐control vector plasmids or HA‐tagged HIF‐1α expression plasmids. Luciferase activity was assessed, as described in the Materials & Methods. Data are normalized to low cellular density‐cultured cells transfected with WT, or HA control vector plasmid‐transfected cells under low cellular density conditions as 100%. Data are the mean ± SD of three or more than three independent experiments. *P < 0.05, *t* test or one‐sample *t* test, significantly different from low cellular density‐cultured cells transfected with WT or mutated human EP4 receptor promoter plasmids. ^†^ *P *< 0.05, *t* test or one‐sample *t* test, significantly different from HA control vector plasmid‐transfected cells under low cellular density conditions. n.s.; not significant

The expression of human EP4 receptors was previously reported to decrease in a cellular density‐dependent manner in HCA‐7 human colon cancer cells, and inversely correlated with HIF‐1α expression levels.^9^, ^16^ One major HIF‐1α‐binding sequence is GCGTG,^24^ namely HRE, which is located between −230 and −226 bp of the human EP4 receptor promoter region (Figures 1A and E). In order to confirm HIF‐1α‐mediated cellular density‐dependent decreases in EP4 receptor expression, deletion mutants of the human EP4 receptor promoter region connected to luciferase reporter genes were constructed and translational activities were assessed, as shown in Figure 1A. As we previously reported,^16^ high cellular density‐cultured HCA‐7 cells transfected with WT (−1238/+1) reporter gene plasmids exhibited approximately 40% lower activities than the corresponding low cellular density‐cultured HCA‐7 cells (Figure 1A, WT). Similar results were obtained in cells transfected with the del 1 (−789/+1) and del 2 (−473/+1) reporter gene plasmids. When HCA‐7 cells were transfected with the del 3 (−197/+1) reporter gene plasmids, which had no HRE region, the total promoter activity of the EP4 receptor promoter decreased under low cellular density conditions; however, the significant high cellular density‐dependent reduction in promoter activity was retained. When cells were transfected with the del 4 (−160/+1) reporter gene plasmids, the cellular density‐dependent reduction in EP4 promoter activity was canceled; therefore, low and high cellular density‐cultured cells exhibited similar promoter activities.

The cellular density‐dependent reduction in EP4 promoter activity was shown to be mediated by increases in the protein expression levels of HIF‐1α.^16^ Therefore, in order to confirm this, HA‐tagged HIF‐1α expression plasmids were transfected into low cellular density‐cultured HCA‐7 cells with the WT (−1238/+1), del 3 (−197/+1), or del 4 (−160/+1) reporter gene plasmids. As shown in Figure 1B and Supporting Information 1B *,* when transfected with HA‐tagged HIF‐1α, WT (−1238/+1) reporter gene plasmid transfected low cellular density‐cultured cells showed similar significant reductions in EP4 receptor promoter activity of approximately 30%‐40% to those of HA‐empty vector plasmid‐transfected control cells, as observed for high cellular density‐cultured cells shown in Figure 1A. Similar results were obtained for HA‐tagged HIF‐1α with the del 3 (−197/+1) reporter gene plasmids in low cellular density‐cultured cells (Figure 1C and Supporting Information 1C). In contrast, when the del 4 (−160/+1) reporter gene plasmids were transfected with the HA‐tagged HIF‐1α expression plasmids shown in Figure 1D and Supporting Information 1D, no significant decrease or increase was observed. Thus, increases in HIF‐1α expression appear to regulate the activation of cellular density‐dependent EP4 receptor promoters acting between −197 and −160 bp.

### 3.2 HRE may not be involved in cellular density‐dependent EP4 receptor promoter activity

Cellular density dependency was also detected in the del 3 (−197/+1) reporter gene plasmids, which lack the HIF‐1α‐binding sequence HRE. In order to examine whether HRE is involved in cellular density‐dependent EP4 receptor promoter activity, point mutations were introduced into the HRE region of WT (−1238/+1) reporter gene plasmids, GCGTG (WT) to GAATG (mut‐HRE),^24^ as shown in Figure 1E. Before investigating the cellular density dependency, the binding ability of mut‐HRE to HIF‐1α was assessed using the ChIP assay. Figure 1F showed that WT, but not mut‐HRE, detected the HIF‐1α‐bound DNA sequence, indicating that mut‐HRE lost its binding ability to HIF‐1α. Cellular density‐dependent EP4 receptor promoter activity was then examined using the mut‐HRE (−1238/+1) reporter gene plasmids. As shown in Figure 1G, mut‐HRE (−1238/+1) reporter gene‐transfected cells did not cancel cellular density dependency; EP4 receptor promoter activity was significantly weaker in high cellular density‐cultured cells than in low cellular density‐cultured cells, similar to WT (−1238/+1) reporter gene‐transfected cells. Therefore, the effects of HA‐tagged HIF‐1α overexpression on EP4 receptor promoter activity were then investigated. As shown in Figure 1H, similar results were obtained in cells transfected with WT (−1238/+1) or mut‐HRE (−1238/+1) reporter gene plasmids under low cellular density conditions. Thus, when transfected with HA‐tagged HIF‐1α, in both WT (−1238/+1) reporter gene plasmids and mut‐HRE (−1238/+1) reporter gene plasmids, showed similar levels of significant reduction of EP4 receptor promoter activity, similar to high cellular density‐cultured cells shown in Figure 1B. These results indicate that HIF‐1α, which directly binds to HRE, may not be involved in cellular density‐dependent EP4 receptor promoter activity.

### 3.3 Sp‐1‐binding motifs are required for cellular density‐dependent EP4 receptor promoter activity

**Figure 2:**
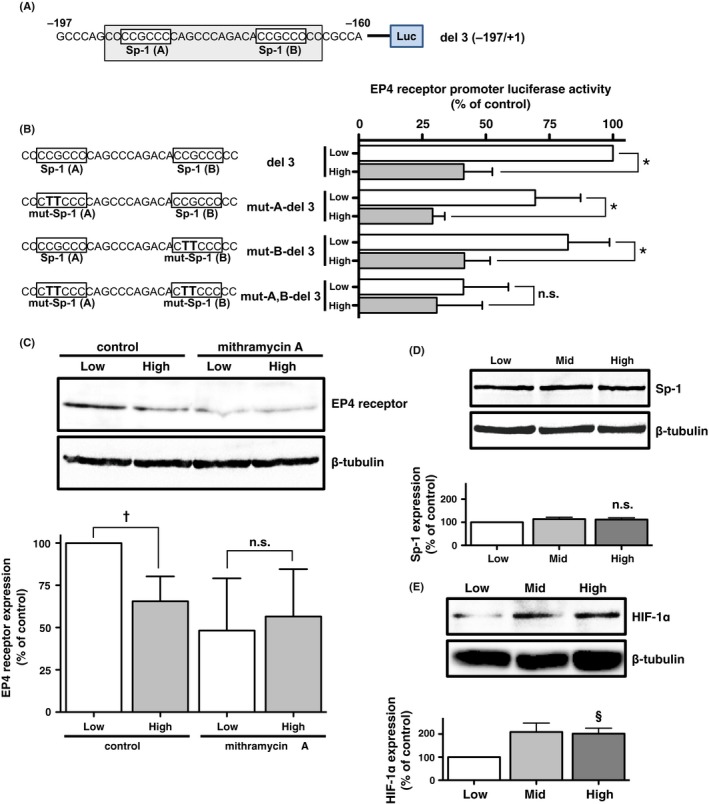
Sp‐1 binding is responsible for the regulation of human EP4 receptor promoter activity. Cells were cultured under low (Low; 2 × 10^5^ cells/each well of 6‐well plate), middle (Mid; 6 × 10^5^ cells/each well of 6‐well plate), and high (High; 2 × 10^6^ cells/each well of 6‐well plate) cellular density conditions in HCA‐7 cells. (A) Two Sp‐1‐binding motifs located between −197 and −160 of the human EP4 receptor promoter region. (B) HCA‐7 cells were transfected with reporter gene plasmids containing del 3 (−197/+1) or each or both Sp‐1‐binding motif‐mutated del 3 plasmids and then subjected to the luciferase assay, as described in the Materials & Methods. Sp‐1‐binding motif mutation maps and the corresponding luciferase activities of del 3 (−197/+1) or each or both Sp‐1‐binding motif‐mutated del 3 plasmids (mut‐A‐del 3, mut‐B‐del 3, and mut‐A,B‐del 3) obtained from HCA‐7 cells cultured under low or high cellular density conditions. (C) An immunoblot analysis with an antibody against EP4 receptors (upper panel) or *β*‐tubulin (lower panel), and a histogram representing the ratio of EP4 receptors to *β*‐tubulin as assessed with pooled densitometric data (mean ± SD) from more than three independent experiments on HCA‐7 cells cultured under low or high cellular density conditions and treated with either vehicle or 100 nmol/L mithramycin A. (D, E) An immunoblot analysis with an antibody against Sp‐1 (D) HIF‐1α (E) (upper panels), or *β*‐tubulin (lower panels), and a histogram representing the ratio of Sp‐1 (D) or HIF‐1α (E) to *β*‐tubulin as assessed with pooled densitometric data (mean ± SD) from three or more than three independent experiments on HCA‐7 cells cultured under low, middle, or high cellular density conditions. Data are normalized to low cellular density‐cultured cells transfected with del 3 (B), or immunoblot ratios of EP4 receptors (C), Sp‐1 (D), or HIF‐1α (E) to *β*‐tubulin detected from low cellular density‐cultured control HCA‐7 cells as 100%. *P < 0.05, *t* test or one‐sample *t* test, significantly different from low cellular density‐cultured cells transfected with WT (B). ^†^ *P *< 0.05 and ^§^ *P *< 0.05, analysis of variance, vs each immunoblot ratio obtained at low cellular density‐cultured cells (C, E). n.s.; not significant

According to the results shown in Figure 1A, a key sequence for the HIF‐1α‐mediated cellular density‐dependent reduction in EP4 receptor promoter activity may be laid on the sequence between −197 and −160 bp. As shown in Figure 2A and as reported previously,^15^ two Sp‐1‐binding motifs, CCGCCC, were detected between −197 to −160 bp of the EP4 promoter region. Therefore, mutations were introduced into each motif and in both motifs of the Sp‐1‐binding sequences. Figure 2B shows the WT and mutated sequences of two Sp‐1‐binding sequences of the del 3 (−197/+1) reporter genes and their corresponding EP4 receptor promoter activities. Although the total activities of low density‐cultured cells transfected with reporter genes slightly decreased when mutations were introduced into one of the two Sp‐1 motifs, mut‐A‐del 3 and mut‐B‐del 3, cellular density‐dependent reductions in activities were retained, similar to the del 3 (−197/+1) reporter gene‐transfected cells. However, when mutations were introduced into both Sp‐1‐binding motifs, mut‐A,B‐del 3, the cellular density dependency of EP4 receptor promoter activity was canceled, similar to the del 4 (−160/+1) in Figure 1A. These results indicate that at least one Sp‐1‐binding motif is required for cellular density‐dependent EP4 receptor promoter activity. Additionally, loss of both Sp‐1 motifs causes a substantial reduction in promoter activity even at low cellular density, as seen also with del 4 (−160/+1).

Based on the involvement of Sp‐1 in the regulation of EP4 receptor expression, we then treated HCA‐7 cells cultured under low and high cellular density conditions with mithramycin A, an antibiotic that binds to the Sp‐1‐binding site to displace Sp‐1 and inhibit its activity. As shown in the left two lanes of Figure 2C, EP4 receptor expression levels were significantly lower by approximately 30%‐40%, in high density‐cultured HCA‐7 cells than in low density‐cultured HCA‐7 cells, and closely correlated with promoter activity, as shown in Figure 1A. However, EP4 receptor expression levels decreased to similar levels in high and low cellular density‐cultured HCA‐7 cells treated with mithramycin A, and cellular density dependency was canceled (Figure 2C, right two lanes). These results indicate that the binding of Sp‐1 to Sp‐1‐binding sites in the EP4 receptor promoter region is directly related to cellular density‐dependent receptor expression.

We previously reported that HIF‐1α protein expression levels increased in a cellular density‐dependent manner with a negative correlation with EP4 receptor expression levels in HCA‐7 cells.^16^ Thus, since Sp‐1 appeared to bind directly to the promoter region of EP4 receptors, the cellular density‐dependent protein expressions of Sp‐1 in HCA‐7 cells were examined. As shown in Figure 2D, no significant differences in Sp‐1 expression were observed among the different cellular densities tested. On the other hand, the expression of HIF‐1α significantly increased in a cellular density‐dependent manner (Figure 2E), similar to that reported previously.^16^

### 3.4 The transcriptional activity of EP4 receptors may be oppositely regulated by HIF‐1α and c‐Myc on the promoter at Sp‐1‐binding sites

**Figure 3:**
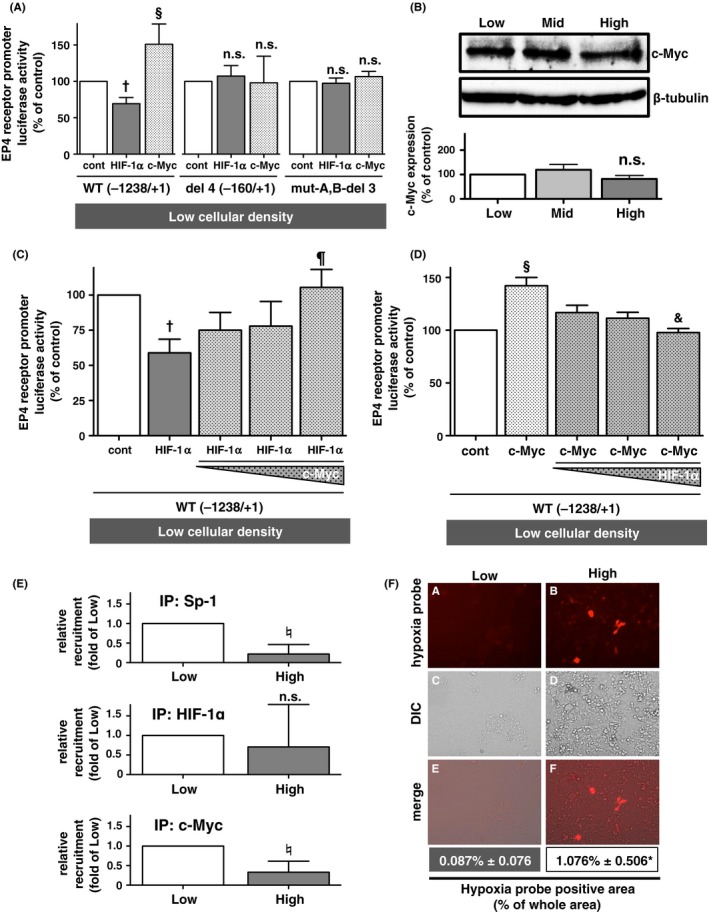
Effects of HIF‐1α and c‐Myc on human EP4 receptor promoter activity in low cellular density‐cultured HCA‐7 cells. Cells were cultured under low (Low; 2 × 10^5^ cells/each well of 6‐well plate), middle (Mid; 6 × 10^5^ cells/each well of 6‐well plate), and high (High; 2 × 10^6^ cells/each well of 6‐well plate) cellular density conditions in HCA‐7 cells. (A) The luciferase activities of HCA‐7 cells cultured under low cellular density conditions and transfected either with WT (−1238/+1), del 4 (−160/+1) or mut‐A,B‐del 3 of EP4 promoter‐containing reporter gene plasmids concomitantly with either HA‐control vector plasmids, HA‐tagged HIF‐1α expression plasmids, or FLAG‐tagged c‐Myc expression plasmids. (B) An immunoblot analysis with an antibody against c‐Myc (upper panel) or *β*‐tubulin (lower panel), and a histogram representing the ratio of c‐Myc to *β*‐tubulin as assessed with pooled densitometric data (mean ± SD) from three or more than three independent experiments in HCA‐7 cells cultured under low, middle, or high cellular density conditions. (C, D) The luciferase activities of HCA‐7 cells cultured under low cellular density conditions transfected with WT (−1238/+1) of EP4 promoter‐containing reporter gene plasmids concomitantly with either HA‐control vector plasmids or HA‐tagged HIF‐1α expression plasmids alone, or HA‐tagged HIF‐1α expression plasmids plus various amounts of FLAG‐tagged c‐Myc expression plasmids (C); or with either HA‐control vector plasmids or FLAG‐tagged c‐Myc expression plasmids alone, or FLAG‐tagged c‐Myc expression plasmids plus various amounts of HA‐tagged HIF‐1α expression plasmids (D). (E) ChIP assay with anti‐Sp‐1, anti‐HIF‐1α and anti‐c‐Myc antibodies performed to verify the binding of Sp‐1, HIF‐1α and c‐Myc to HRE point mutated WT (mut‐HRE (−1238/+1)) of the EP4 promoter in HCA‐7 cells cultured under low and high cellular density conditions. (F) HCA‐7 cells were cultured under low (Low; 2 × 10^5^ cells/each well of 6‐well plate) or high (High; 2 × 10^6^ cells/each well of 6‐well plate) cellular density conditions, and the hypoxia probe solution was then added to the medium in order to assess hypoxic areas, as described in the Materials & Methods. Picture panels (A‐F) were from a representative experiment that was repeated three times. Data are normalized to low cellular density‐cultured HCA‐7 cells transfected with control vector plasmid‐transfected cells (A, C, D), or immunoblot ratio of low cellular density‐cultured cells (B) as 100%. Data are the mean ± SD of three or more than three independent experiments. *P < 0.05, *t* test, significantly different from the hypoxia probe‐positive area of low cellular density‐cultured cells. ^†^ *P *< 0.05, analysis of variance, vs HA control vector plasmid‐transfected cells under low cellular density conditions. ^§^ *P *< 0.05, analysis of variance, vs HA control vector plasmid‐transfected cells under low cellular density conditions. ^¶^ *P *< 0.05, analysis of variance, vs the results of HA‐tagged HIF‐1α expression plasmid‐transfected cells under low cellular density conditions. ^&^ *P *< 0.05, analysis of variance, vs the results of FLAG‐tagged c‐Myc expression plasmid‐transfected cells under low cellular density conditions. ^♮^ *P *< 0.05, one‐sample *t* test, significantly different from the each plasmid‐transfected cells under low cellular density conditions. DIC; differential interference contrast, n.s.; not significant

Previous studies reported that HIF‐1α interacts with Sp‐1, which binds to the promoter lacking the HRE region.^21^, ^22^ Moreover, HIF‐1α has been shown to displace c‐Myc from the promoter binding Sp‐1, resulting in the repression of its promoter activity, that is, the MSH2 promoter of human sporadic colon cancer cells.^21^, ^22^ Thus, in order to examine whether cellular density‐dependent EP4 receptor promoter activities are regulated by HIF‐1α and/or c‐Myc through promoter‐bound Sp‐1, HIF‐1α and c‐Myc proteins were overexpressed in low cellular density‐cultured HCA‐7 cells. Figure 3A and Supporting Information 3A show that when transfected with WT (−1238/+1) reporter gene plasmids, HIF‐1α‐overexpressing cells exhibited significantly reduced EP4 receptor promoter activity, similar to that shown in Figure 1H. In contrast, EP4 receptor promoter activity was significantly stronger in c‐Myc overexpressing cells than in vector control‐transfected cells (Figure 3A, left columns). Furthermore, when transfected with the del 4 (−160/+1) or mut‐A,B‐del 3 reporter gene plasmids, which lack or mutated two Sp‐1‐binding regions respectively, these HIF‐1α‐mediated inhibitory and c‐Myc‐mediated stimulatory effects were abolished (Figure 3A, middle and right columns). These results indicate that the transcriptional activity of EP4 receptors is oppositely regulated by HIF‐1α and c‐Myc on the promoter at Sp‐1‐binding sites. As a comparison, when the cellular density‐dependent protein expression of c‐Myc in HCA‐7 cells was examined, no significant differences were observed in c‐Myc expression among the different cellular densities tested (Figure 3B).

Based on the opposite effects exerted by HIF‐1α and c‐Myc, competition assays were performed in order to clarify whether increases in HIF‐1α or c‐Myc reduce the effects of its counterpart. As shown in Figure 3C and Supporting Information 3C, HIF‐1α‐overexpressing cells exhibited significantly reduced WT (−1238/+1) reporter gene promoter activity, similar to that shown in Figures 1H and 3A. However, in HCA‐7 cells cotransfected with c‐Myc plasmid, the suppressed promoter activity recovered to the same levels as the control cells depending on the amount of the c‐Myc plasmid. Conversely, in c‐Myc‐overexpressing HCA‐7 cells cotransfected with HIF‐1α, the enhanced promoter activity declined to the same levels as the control cells depending on the amount of the HIF‐1α plasmid (Figure 3D and Supporting Information 3D). Finally, to confirm the cellular density‐dependent recruitment of each Sp‐1, HIF‐1α, and c‐Myc to the Sp‐1‐binding sites was analyzed by ChIP assay. As shown in Figure 3E, the significant cellular density‐dependent decrease in binding ability to mut‐HRE (−1238/+1) to Sp‐1 and c‐Myc, but not to HIF‐1α, indicating that promoter region of EP4 receptor lost its binding ability to Sp‐1 and/or c‐Myc when cells were cultured in high cellular density.

### 3.5 High cellular density‐cultured HCA‐7 cells showed significantly larger hypoxia‐positive area than in low cellular density‐cultured cells

As shown in Figures 2D, E, and 3B, the protein expression levels of Sp‐1 and c‐Myc were not altered in a cellular density‐dependent manner, in contrast to HIF‐1α. Hypoxia is one of the critical factors inducing HIF‐1α, which is frequently overexpressed in many cancers including colon cancer.^17^, ^18^, ^19^ Thus, in order to confirm whether the cellular density‐dependent induction of HIF‐1α was due to hypoxic cellular conditions, hypoxic areas were measured using the hypoxia probe. As shown in Figure 3F, the hypoxia‐positive area in low cellular density‐cultured HCA‐7 cells was approximately 0.087% whereas that in high cellular density‐cultured cells was approximately 1.076%, an area that was approximately 10‐fold more significant than that in low cellular density‐cultured cells. Thus, elevations in hypoxia under high cellular density conditions are a plausible reason for the up‐regulated expression of HIF‐1α. Although the hypoxia‐positive area was approximately 1% of the total area, the high density‐cultured HCA‐7 cells were in sphere‐like/multilayers phase, so that there was a possibility that the hypoxia probe might not penetrate all the way down to the underlying and/or bottom cells. Therefore, even the majority of the cells did not appear to be hypoxia‐positive, it was difficult to conclude that the bulk of the HCA‐7 cells were not in hypoxia.

### 3.6 HIF‐1α protein levels may increase with decreases in the activity of its degradation pathways, which induce the HIF‐1α‐mediated down‐regulation of EP4 receptor mRNA expression

**Figure 4:**
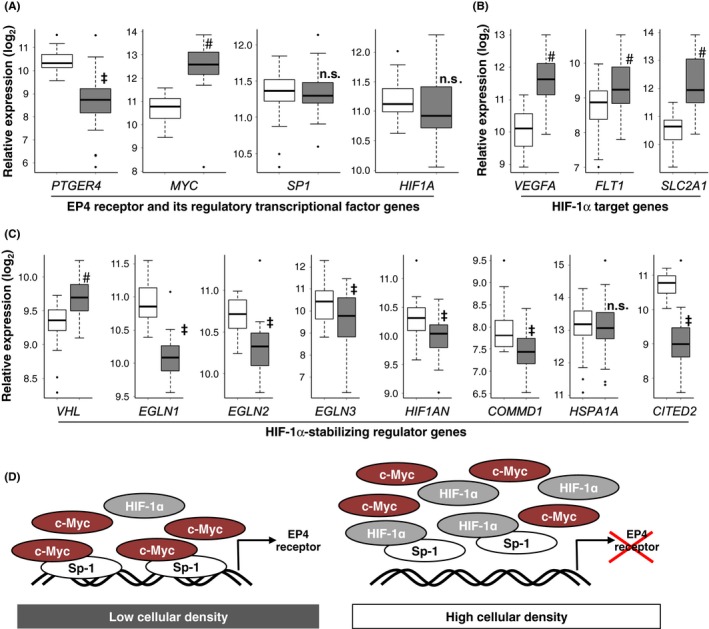
EP4 receptor and HIF1α‐related gene expression in human colon and rectal cancer tissues analyzed using the TCGA database. (A, B, C) Comparison of the mRNA expression of EP4 receptors (PTGER4), c‐Myc (MYC), Sp‐1 (SP1), and HIF‐1α (HIF1A) (A), HIF‐1α target mRNAs such as VEGF‐A_165_ (VEGFA), VEGFR‐1 (FLT1), and GLUT‐1 (SLC2A1) (B), and the factors involved in the stabilization of HIF‐1α such as von Hippel‐Lindau (VHL), prolyl hydroxylase domain‐containing enzymes (PHDs; also known as Egl‐9 family hypoxia‐inducible factors [EGLNs]), factor‐inhibiting HIF‐1α (FIH1; also known as HIF‐1α inhibitor [HIF1AN]), copper metabolism domain‐containing 1 (COMMD1), heat shock protein (HSP) 70 proteins, such as HSPA1A, and cAMP response element‐binding protein‐binding protein (CBP)/p300‐interacting transactivator 2 (CITED2) (C), between cancer tissues (gray boxes) and paired noncancer tissues (white boxes). ^&^ *P *< 0.05, the Mann‐Whitney *U*‐test, significantly lower than noncancer tissues. ^#^ *P *< 0.05, the Mann‐Whitney *U*‐test, significantly higher than noncancer tissues. n.s.; not significant (D) Schematic models depict the c‐Myc‐Sp‐1 complex‐mediated transcriptional activation of EP4 receptors in low cellular density‐cultured HCA‐7 cells. In high cellular density‐cultured HCA‐7 cells, increased HIF‐1α competes with and displaces c‐Myc for Sp‐1 binding and followed by pulling Sp‐1 out from its binding site, resulting in the down‐regulation of EP4 receptor transcriptional activation

In order to confirm the relevance of these results, an in silico analysis was performed using the Cancer Genome Atlas database; http://xena.ucsc.edu.^25^ As shown in Figure 4A, the expression levels of EP4 receptor mRNAs were significantly lower in colorectal cancer tissues (gray boxes) than in the corresponding normal tissues (white boxes). In contrast, the expression levels of c‐Myc mRNAs were significantly higher in colorectal cancer tissues than in normal tissues. The expression levels of Sp‐1 as well as HIF‐1α mRNAs were similar, with no significant differences being observed between cancer and normal tissues.

We previously reported that reductions in EP4 receptors in high cellular density‐cultured HCA‐7 cells by the induction of HIF‐1α switched the responsible primary EP receptor subtypes from EP4 receptors to stationary EP3 receptors.^9^, ^16^ We also demonstrated that the stimulation of EP3 receptors induced vascular endothelial growth factor (VEGF)‐A_165_ (VEGF‐A) and VEGF receptor‐1 (VEGFR‐1, also known as FLT‐1) in HCA‐7 cells and HEK‐293 cells stably expressing human EP3 receptors.^26^, ^27^ Thus, as shown in Figure 4B, the expression levels of VEGF‐A and FLT‐1 mRNAs were significantly higher in cancer tissues (gray boxes) than in normal tissues (white boxes). Although we have not yet proven whether increases in the induction of VEGF‐A and FLT‐1 mRNAs in cancer tissues, as shown in Figure 4B, are EP3 receptor‐mediated events, the expression of these angiogenic‐related factors is known to be regulated by HIF‐1α.^28^ Another well‐known example of HIF‐1α‐regulated mRNA expression is glucose transporter (GLUT)‐1 (also known as SLC2A1) mRNA, because the transport of glucose plays essential roles in the development of embryos in the relatively hypoxic environment of the placenta.^29^ As shown in Figure 4B, the mRNA expression levels of SLC2A1 were also significantly higher in cancer tissues (gray box) than in normal tissues (white box). The stabilized protein expression levels of HIF‐1α are also regulated by degradation rates through the ubiquitin‐proteasome system under normoxic conditions.^28^ Thus, the protein expression levels of HIF‐1α shown in Figure 2E may be increased regardless of mRNA levels by inhibiting the degradation pathways. Several factors are involved in the stabilization of HIF‐1α such as the von Hippel‐Lindau (VHL) protein, prolyl hydroxylase domain‐containing enzymes (PHDs; also known as Egl‐9 family hypoxia‐inducible factors (EGLNs)), factor‐inhibiting HIF‐1α (FIH1; also known as HIF‐1α inhibitor (HIF1AN)), copper metabolism domain‐containing 1 (COMMD1), heat shock protein (HSP) 70 proteins, such as HSPA1A, and cAMP response element‐binding protein‐binding protein (CBP)/p300‐interacting transactivator 2 (CITED2).^28^ Figure 4C shows comparisons of mRNAs involved in HIF‐1α stabilization between cancer (gray boxes) and normal (white boxes) tissues as listed above. Except for VHL and HSPA1A, six out of eight mRNAs were significantly decreased in cancer tissues, which is consistent with widely accepted view that the degradation pathway is primarily suppressed under hypoxic condition in cancer tissue.^17^ Therefore, even though no significant differences were observed in HIF‐1α mRNA expression levels between cancer and normal tissues (Figure 4A), HIF‐1α protein levels may have increased due to decreases in the activity of its degradation pathways (Figure 4C), which significantly induced HIF‐1α‐mediated VEGF‐A, FLT‐1, and SLC2A1 mRNA expression (Figure 4B), as well as the reduction of the EP4 receptor expressions (Figure 4A).

## 4 DISCUSSION

### 4.1 HRE‐bound HIF‐1α may be responsible for positively regulating basal EP4 receptor promoter activity

The up‐regulated expression of EP4 receptors has been implicated in carcinogenesis.^5^, ^6^ However, the expression levels of EP4 receptors were also found to be significantly lower in tumor tissues than in normal tissues.^14^ We previously demonstrated that EP4 receptor expression levels might be altered in a cellular density‐dependent manner.^16^ Thus, the cellular density‐dependent down‐regulation of EP4 receptors was previously shown to be regulated via the up‐regulation of HIF‐1α in HCA‐7 cells.^16^ In the present study, we elucidated the underlying mechanisms by which HIF‐1α down‐regulates EP4 receptor expression.

Although the existence of the HRE site, to which HIF‐1α binds directly, was confirmed in the EP4 promoter region (Figures 1E and F), HRE was not responsible for the cellular density‐dependent down‐regulation of EP4 receptor expression. Since the knockdown of HIF‐1α by siRNA altered the cellular density‐dependent down‐regulation of EP4 receptors as shown previously,^16^ HIF‐1α is a crucial factor for this regulation. Whereas, as shown in Figures 1G and H, mutation‐induced HRE, which did not bind to HIF‐1α (Figure 1F), exerted negligible effects on cellular density dependency. However, based on the results shown in Figure 1A, when basal EP4 receptor promoter activities in low density‐cultured cells were compared between del 2 and del 3 mutant‐transfected cells, del 3‐transfected cells showed markedly weaker basal activity while retaining cellular density dependency. Surprisingly, there are only two transcriptional factor‐binding motifs in EP4 receptor promoter region between −472 (del2) to −197 (del3), analyzed by JASPAR ( http://jaspar.genereg.net/) using “CORE” collection. Thus, one is HRE (−230 to −226) and the other is a BARHL2‐binding site (−302 to −293). Since BARHL2 is initially cloned and characterized from the central nervous system,^30^ and the mRNA of BARHL2 is only expressed in testis (NCBI Gene ID: 343472),^31^ we therefore, believe HRE is the principal transcriptional factor‐binding motif between −472 to −197 in human EP4 receptor promoter region. However, the possibility that there is other modulation protein(s) and/or binding motif(s) involved in transcription efficiency that is not included in the “CORE” collection cannot be ruled out. Moreover, the basal promoter activities were slightly weaker in mut‐HRE (−1238/+1) plasmid‐transfected cells than in WT (−1238/+1) plasmid‐transfected cells cultured under low cellular density conditions (Figures 1G and H). Thus, in contrast to Sp‐1‐bound HIF‐1α, HRE‐bound HIF‐1α may be responsible for positively regulating basal EP4 receptor promoter activity.

### 4.2 HIF‐1α and c‐Myc may be critical factors for maintaining the homeostasis of colorectal epithelial cells by regulating EP4 receptor expression

As described, while EP4 receptors are generally known to be involved in colorectal carcinogenesis, they have also played a role in maintaining gastrointestinal homeostasis.^10^ Thus, during the 3‐5 days turnover of epithelial cells, *β*‐catenin‐mediated signaling is activated for proliferation and migration in the first half, followed by its inactivation for differentiation and apoptosis in the last half. Since EP4 receptors are known to activate *β*‐catenin‐mediated signaling,^20^ the proliferation and migration of colorectal epithelial cells appear to be mediated by the activation of EP4 receptor‐expressing cells in the first of the cycle; whereas, in the last half of the cycle, *β*‐catenin‐mediated signaling is inhibited for differentiation and apoptosis,^13^ and the present results may explain the underlying mechanisms. Thus, due to the up‐regulation of proliferation by EP4 receptor‐activated *β*‐catenin‐mediated signaling, HIF‐1α protein levels may also be up‐regulated. However, when cells reach the midcrypt region, the accumulation of HIF‐1α may inhibit *β*‐catenin‐mediated signaling, following to the down‐regulation of EP4 receptor expression by displacing c‐Myc for Sp‐1 binding at the receptor promoter region and followed by pulling Sp‐1 out from its binding site (Figure 4D), since HIF‐1α was shown not to bind mut‐HRE (Figure 1F), as well as no cellular density‐dependent decrease in the binding ability was observed (Figure 3E). Therefore, HIF‐1α may be a critical factor in maintaining the homeostasis of colorectal epithelial cells by initiating a negative feedback loop of EP4 receptor expression. Another key factor for controlling the fate of cells by changing EP4 receptor expression levels may be the activity of c‐Myc. As shown in Figures 3A and C, the overexpression of c‐Myc up‐regulated EP4 receptor transcriptional activities. The expression of c‐Myc is known to be positively regulated by *β*‐catenin‐mediated signaling.^32^ Since *β*‐catenin‐mediated signaling is regulated by the activation of EP4 receptors, c‐Myc may initiate a positive feedback loop of EP4 receptor expression.

The 3‐5 days cycle of homeostasis of normal colorectal epithelial cells appears to be positively regulated by the expression levels of EP4 receptors through c‐Myc in the first half, and then negatively regulated via HIF‐1α in the last half. However, since this normal homeostasis cycle is rapid, once the balance between c‐Myc‐ and HIF‐1α‐mediated regulation become altered, these epithelial cells may easily become cancerous due to aberrant proliferation, migration, and differentiation. When epithelial cells reach the midcrypt region, normal cells inhibit *β*‐catenin‐mediated signaling for differentiation and apoptosis; however, if c‐Myc is strongly expressed, *β*‐catenin‐mediated signaling remains to activate in progenitor cells in the first half of the cycle, which leads to aberrant proliferation and, ultimately, a cancerous phenotype.^13^ As shown in Figure 4A, the induction of c‐Myc mRNA was significantly stronger in cancer tissues than in normal tissues, and the overexpression of c‐Myc may be one of the first steps in carcinogenesis, which may be because of the overexpression of EP4 receptors.^5^, ^6^ Furthermore, an increase in the induction of c‐Myc was not detected in HCA‐7 cells, as shown in Figure 3B. This may have been because HCA‐7 cells retain some features of normal colon epithelial cells.^33^ However, it is more likely that HCA‐7 cells are cancer cells in which c‐Myc may already be overexpressed to nearly maximal levels.

### 4.3 Transforming mechanisms of EP4 receptor expression in homeostasis to cancer development

It currently remains unclear why the expression of EP4 receptors is concomitantly reduced with increases in cancer cell proliferation if these receptors are involved in colorectal cancer malignancy. The present results indicate that EP4 receptors are key receptors for the initiation of carcinogenesis, but the only function at the very early stage of the disease. Thus, if the expression levels of EP4 receptors in normal epithelial cells increase for some reason, the expression levels of c‐Myc will also become elevated through the enhanced activation of *β*‐catenin‐mediated signaling. However, if the cells proliferate rapidly and become cancerous, the expression levels of EP4 receptors are reduced via rapidly and/or aberrantly increased HIF‐1α protein levels, although reductions in receptor expression levels may be facilitated by the same regular homeostasis mechanism. Thus, at the stage of HIF‐1α abundance in cells, EP4 receptors do not appear to be involved in cancer development because the abundance of HIF‐1α also induces VEGF‐A_165_ and VEGFR‐1, as shown in Figure 4B, to provide nutrients for rapidly growing cancer cells through the inductions of angiogenesis and/or cellular migration/metastasis, possibly via the activation of EP3 receptors, as we reported previously.^26^, ^27^ Thus, at the stage of HIF‐1α abundance in colorectal epithelial cells, cells appear to progress to cancerous phenotypes and EP4 receptor‐mediated homeostasis is no longer required.

## 5 CONCLUSIONS AND PERSPECTIVES

Although we could not show the “direct” competition between HIF‐1α and c‐Myc on Sp‐1 transcriptional factor, when the cellular switch for maintaining homeostasis mediated by c‐Myc and HIF‐1α for the Sp‐1‐binding balance is altered, such as the overexpression of EP4 receptors, the cells continue to grow aberrantly and become cancerous phenotypes. EP4 receptors appear to be required for the first step of carcinogenesis, as we have discussed previously,^34^ they no longer have a role once cells have aberrantly proliferated. However, the reduction of expression of EP4 receptors in the bulk of high cellular density‐cultured HCA‐7 cells may have nonstem cell‐like character and there is a possibility that much rare stem cell‐like population may retain the high levels of expressions of EP4 receptors, which could be important for the late stage of cancer progression. Further studies are needed to characterize those studies by using not only HCA‐7 cells (Dukes stage B) but also other types of colon cancer such as DLD‐1 cells and HCT‐15 cells (Dukes stage C). Also the effects of another transcriptional factor, early growth response factor, Egr‐1, should be examined in the future since its binding motif is overlapping to the Sp‐1 site, and Egr‐1 expression would be regulated by cellular density.^35^ However, the present results provide one plausible reason for why conflicting findings exist for the roles of the expression levels of EP4 receptors in carcinogenesis.

## AUTHOR CONTRIBUTIONS

NS carried out introducing mutations to the reporter vectors, luciferase assays, ChIP assay, western blotting, and cell imaging of hypoxia probe assays. YA, NK, and NY carried out luciferase assays and western blotting. KF carried out in silico computer analysis. KY, MM, HN, JWR, and TM participated in the coordination of the experiments and draft manuscript writing. HF conceived, coordinated, designed, and analyzed the experiments as well as manuscript writing.

## DISCLOSURE

The authors declare no conflict of interest.
