# Supplementary material for: Cellular density‐dependent increases in HIF‐1α compete with c‐Myc to down‐regulate human EP4 receptor promoter activity through Sp‐1‐binding region
Source: Pharmacol Res Perspect. 2018 Nov 11;6(6):e00441. doi: 10.1002/prp2.441 (PMC6230926; doi:10.1002/prp2.441)

Supporting information 1B

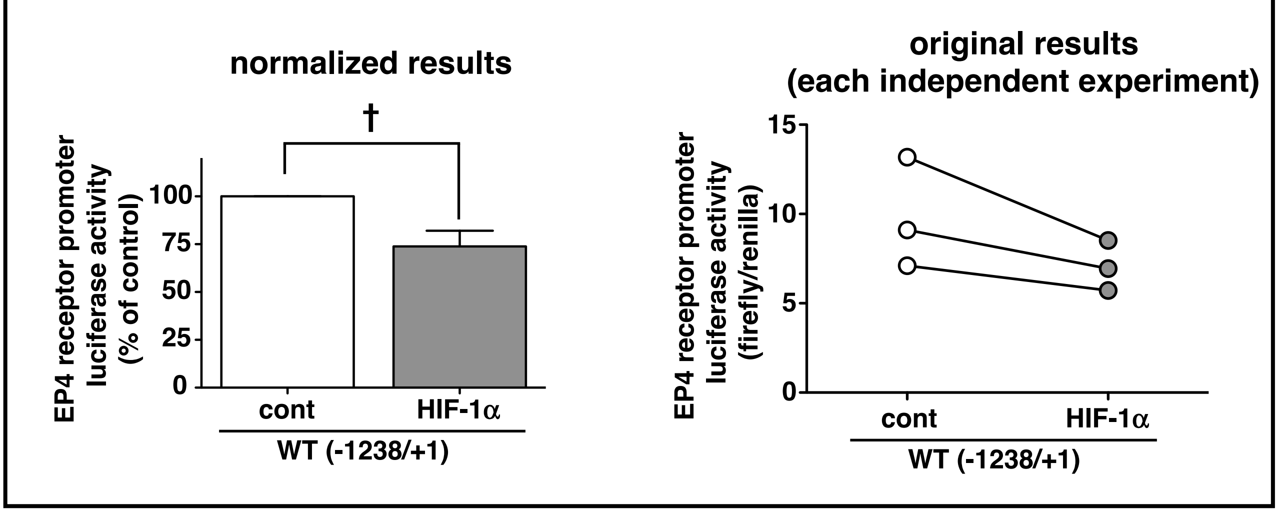

Supporting information 1C

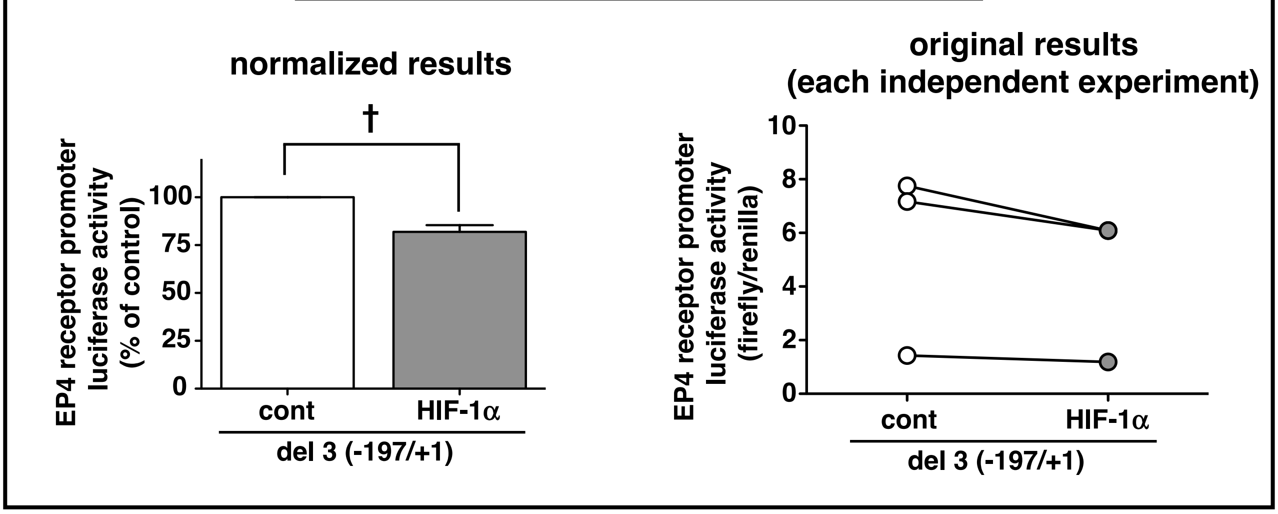

Supporting information 1D

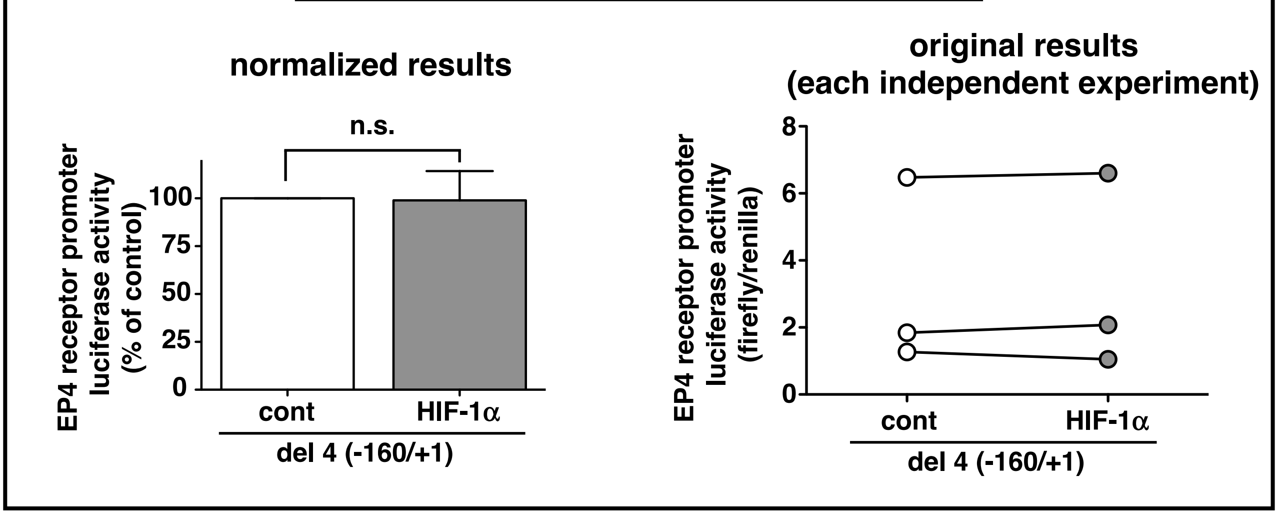

Supporting information 3A (WT)

normalized results

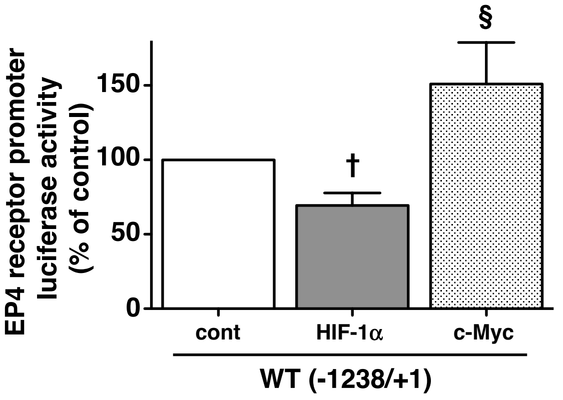

original results (each independent experiment)

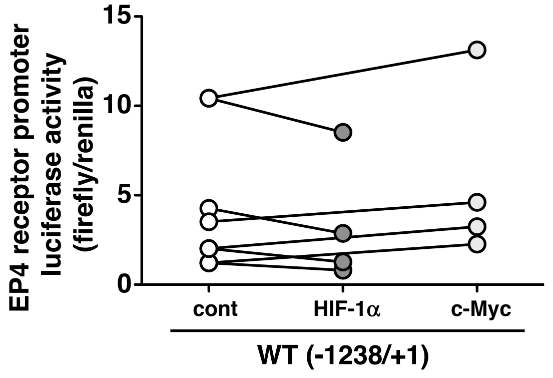

Supporting information 3A (del 4)

normalized results

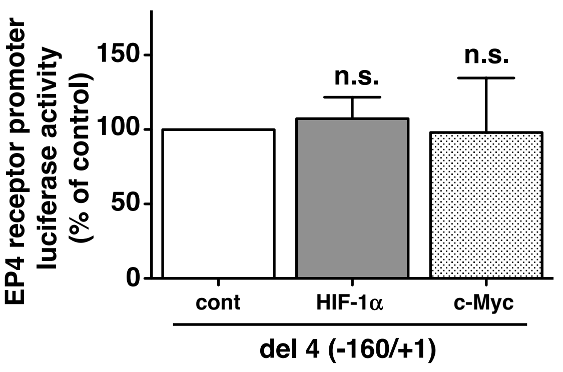

original results (each independent experiment)

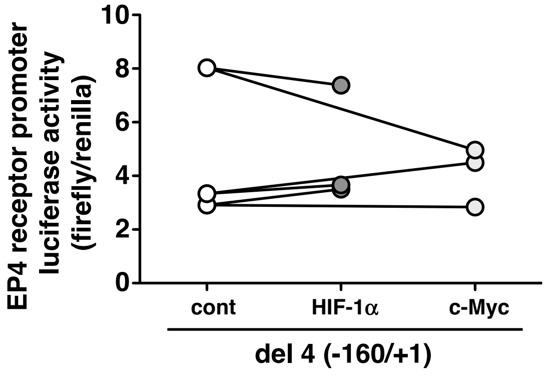

Supporting information 3A (mut-A,B-del 3)

normalized results

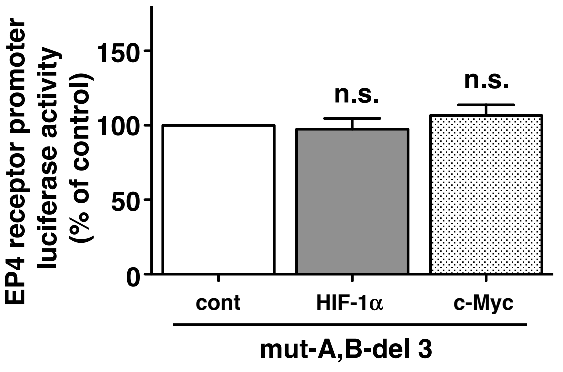

original results (each independent experiment)

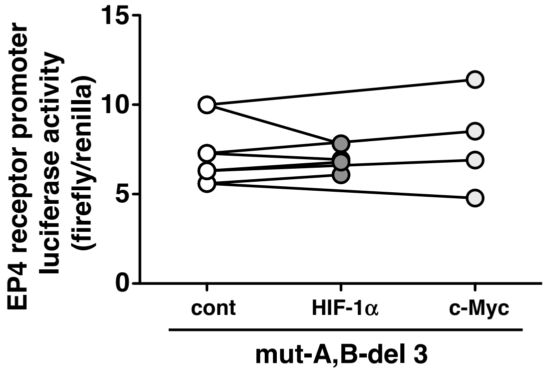

Supporting information 3C

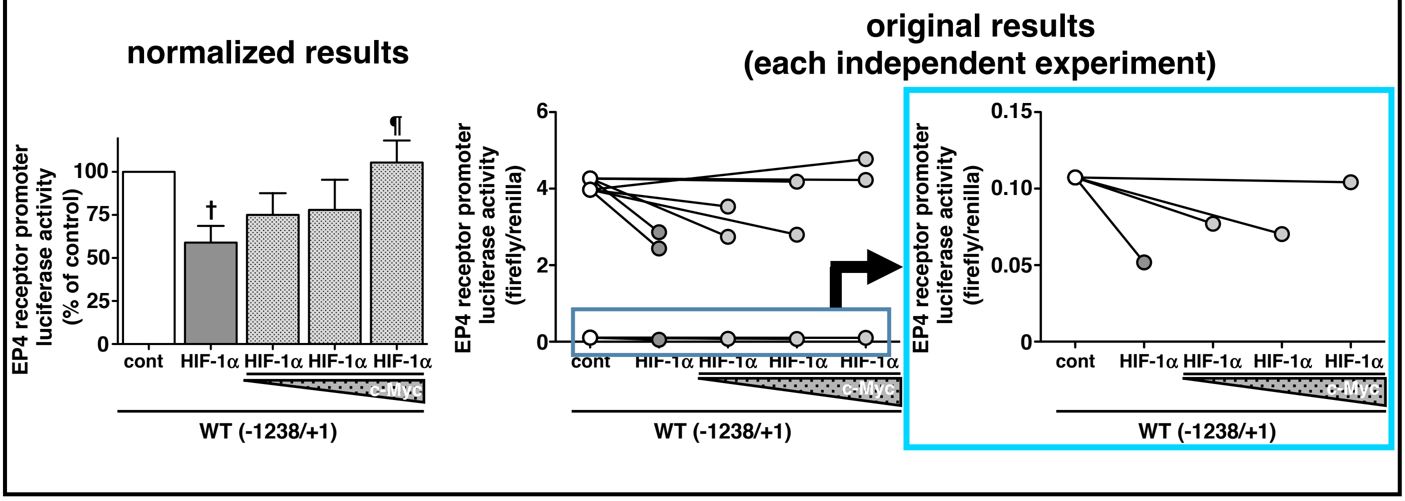

Supporting information 3D

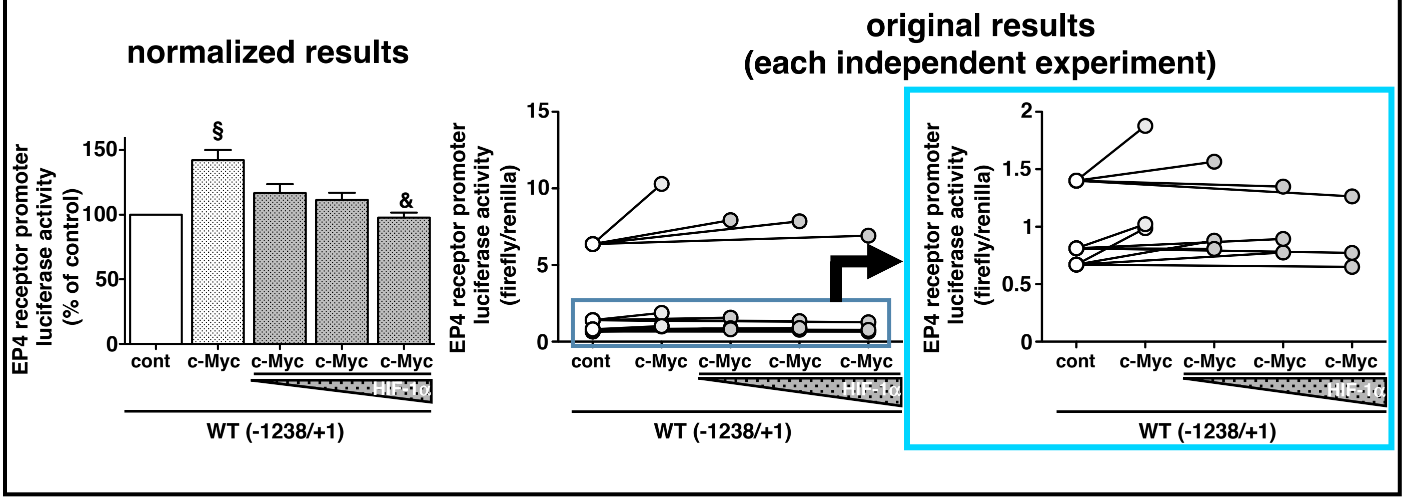

Supplement: Supplementary file 1 [file PRP2-6-e00441-s001.pdf]
